# Beating Heart Transplant Procedures Using Organs From Donors With Circulatory Death

**DOI:** 10.1001/jamanetworkopen.2024.1828

**Published:** 2024-03-11

**Authors:** Aravind Krishnan, Chawannuch Ruaengsri, Brandon A. Guenthart, Yasuhiro Shudo, Hanjay Wang, Michael R. Ma, John Ward MacArthur, William Hiesinger, Y. Joseph Woo

**Affiliations:** 1 Department of Cardiothoracic Surgery, Stanford University School of Medicine, Stanford, California; 2 Department of Bioengineering, Stanford University School of Engineering, Stanford, California

## Abstract

**Importance:**

The use of ex vivo normothermic organ perfusion has enabled the use of deceased after circulatory death (DCD) donors for heart transplants. However, compared with conventional brain death donation, DCD heart transplantation performed with ex vivo organ perfusion involves an additional period of warm and cold ischemia, exposing the allograft to multiple bouts of ischemia reperfusion injury and may contribute to the high rates of extracorporeal membrane oxygenation usage after DCD heart transplantation.

**Objective:**

To assess whether the beating heart method of DCD heart transplantation is safe and whether it has an acceptable rate of extracorporeal membrane oxygenation use postoperatively.

**Design, Setting, and Participants:**

This case series includes 10 patients with end-stage heart failure undergoing DCD heart transplantation at a single academic medical center from October 1, 2022, to August 3, 2023. Data were analyzed from October 2022 to August 2023.

**Interventions:**

Using a beating heart method of implantation of the donor allograft.

**Main Outcomes and Measures:**

The main outcome was primary graft dysfunction necessitating postoperative initiation of mechanical circulatory support. Survival and initiation of mechanical circulatory support were secondary outcomes.

**Results:**

In this case series, 10 consecutive patients underwent DCD heart transplantation via the beating heart method. Ten of 10 recipients were male (100%), the mean (SD) age was 51.2 (13.8) years, and 7 (70%) had idiopathic dilated cardiomyopathy. Ten patients (100%) survived, and 0 patients had initiation of extracorporeal membrane oxygenation postoperatively. No other mechanical circulatory support, including intra-aortic balloon pump, was initiated postoperatively. Graft survival was 100% (10 of 10 patients), and, at the time of publication, no patients have been listed for retransplantation.

**Conclusions and Relevance:**

In this study of 10 patients undergoing heart transplantation, the beating heart implantation method for DCD heart transplantation was safe and may mitigate ischemia reperfusion injury, which may lead to lower rates of primary graft dysfunction necessitating extracorporeal membrane oxygenation. These results are relevant to institutions using DCD donors for heart transplantation.

## Key Points

**Question:**

Is the beating heart method of deceased after circulatory death (DCD) heart transplant a safe method of avoiding additional warm and cold ischemic periods?

**Findings:**

This case series of 10 consecutive patients undergoing heart transplantation from DCD donors found that using a beating heart method avoided a second cardioplegic arrest, which is typically associated with DCD heart transplantation. No patients required initiation of any form of mechanical circulatory support postoperatively.

**Meaning:**

These findings suggest the beating heart method avoided a second cardioplegic arrest and may have helped reduce the high extracorporeal membrane oxygenation rates, which are seen after a DCD heart transplantation.

## Teaser

This case series assesses the safety of the beating heart method of deceased after circulatory death heart transplantation is safe, and if it has an acceptable rate of extracorporeal membrane oxygenation used postoperatively.

## Introduction

The use of donation after circulatory death (DCD) for heart transplantation has expanded the donor pool with comparable outcomes with the reference standard of donation after brain death (DBD) heart transplantation.^1,2,3^ Conventional DCD heart transplantation is facilitated by normothermic ex vivo organ perfusion, whereby the heart is explanted from the donor, and perfused on a perfusion platform.^4,5^ Typically, this requires 2 cardioplegic arrests, compared with DBD which involves only 1 cardioplegic arrest (Figure). Two arrests involve 2 periods of warm ischemia and 2 periods of cold ischemia, exposing the allograft to multiple bouts of ischemia reperfusion injury, potentially contributing to the 40% incidence of extracorporeal membrane oxygenation (ECMO) for primary graft dysfunction after DCD heart transplantation reported in major series.^6^

**Figure.**
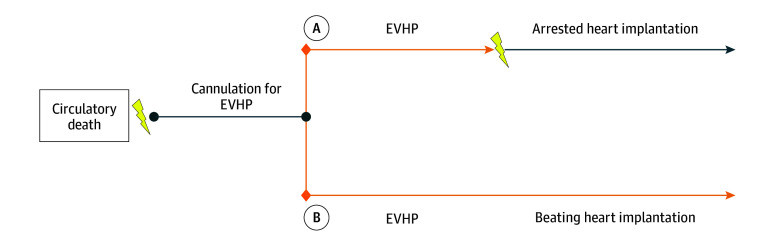
Ischemic Periods in Deceased After Circulatory Death (DCD) Heart Transplantation Path A shows the conventional approach to DCD heart transplantation via ex vivo heart perfusion (EVHP), in which there are 2 cardioplegic arrests and 2 warm ischemic periods, and 2 cold ischemic periods. Each reperfusion events prompts a cycle of ischemia reperfusion injury. The lightning bolts represent ischemic injury. Path B shows the modification to DCD heart transplantation using simultaneous cardiopulmonary bypass and machine perfusion to the allograft while on an EVHP platform. Implantation occurs with the heart beating, without any interruption in perfusion, which removes the additional warm and cold ischemia times.

We have developed a method of performing DCD heart transplantation with uninterrupted coronary perfusion after explanting the heart from the donor, and keeping the heart beating through implantation (Figure).^7^ Our novel beating heart method avoids the second cardioplegic arrest and, with it, the additional warm and cold ischemic periods. There remains only the functional warm ischemic period while the donor is agonal, followed by a single period of cold ischemia while the organ is placed on an ex vivo organ perfusion platform. Thereafter, perfusion remains uninterrupted. To our knowledge, we present the first cases series of patients who underwent DCD heart transplantation via a beating heart implantation method.

## Methods

This case series was deemed exempt from approval by the Stanford University institutional review board. The exemption for the data use for this secondary analysis did not require informed consent. This study follows the reporting guideline for case series.

This was a single-center case series of 10 consecutive patients with end-stage heart failure who underwent DCD heart transplantation at our institution between October 1, 2022, and August 31, 2023. Inclusion criteria included all adult patients at Stanford University School of Medicine listed for heart transplantation for whom a suitable DCD donor was identified and accepted. Suitable donors included those identified by the Maastricht Category III criteria,^8^ as well as extended criteria donors, which includes being older than 50 years, having a size mismatch determined by a greater than 10% difference in predicted heart mass, presence of left ventricular hypertrophy, ejection fraction less than 50%, and history of diabetes.^9^ Exclusion criteria included any concomitant cardiac surgical procedure, such as total arch replacement at the time of heart transplant or multiorgan transplantation.

Reports of race are self-reported by patients and donors. Race was reported per United Network of Organ Sharing convention when considering reporting of donor and recipient demographic data. Race included American Indian or Alaskan Native, Asian, Black, Hispanic or Latino, Native Hawaiian and Other Pacific Islander, White, and multiracial.

Each patient underwent heart transplantation via a beating heart implantation method, whereby there was uninterrupted coronary perfusion starting from initiation of ex vivo organ perfusion through implantation. This was achieved by simultaneously cannulating the donor heart for coronary perfusion from the recipient cardiopulmonary bypass circuit while the allograft was perfused on an ex vivo normothermic perfusion platform and then sewing the graft in while beating (eFigure in Supplement 1). The outcome of interest was postoperative initiation of extracorporeal membrane oxygenation for graft dysfunction, with secondary outcomes of survival, and initiation of mechanical circulatory support, such as intra-aortic balloon pump (IABP) or right ventricular assist device (RVAD).

### Data Analysis

Data analyses were descriptive and calculations were performed Stata version 16.1 (StataCorp). Analysis were performed from January 2023 to December 2023.

## Results

Ten patients were included in this case series (Table). Ten of 10 recipients were male (100%), the mean (SD) age was 51.2 (13.8) years, and 1 American Indian or Alaskan Native patient (10%), 1 Hispanic patient (10%), 1 multiracial patient (10%), and 7 White patients (70%). The most common diagnosis was idiopathic dilated cardiomyopathy (7 [70%]). Two patients (20%) were receiving ECMO preoperatively, 5 (50%) had IABPs in place, and 2 (20%) had a left ventricular assist device (LVAD) in place. Three (30%) patients underwent redo sternotomy, with 2 (67%) for LVAD explant, and 1 (33%) for history of prior aortic valve replacement. Six patients (60%) were listed as status 2, and 2 (20%) were listed as status 1A. Donors were mostly male (9 [90%]) and White individuals (7 [70%]). Two donors (20%) were considered extended criteria; one because of a history of diabetes and the other because of left ventricular hypertrophy on echocardiography.

**Table.** Recipient and Donor Demographics

Characteristic	Participant, No. (%)	
	Recipient	Donor
Age, mean (SD), y	51.2 (13.8)	30.8 (8.9)
Sex		
Male	10 (100)	9 (90.0)
Female	0	1 (10.0)
Race		
American Indian or Alaska Native	1 (10.0)	0
Asian	0	1 (10.0)
Hispanic or Latino	1 (10.0)	2 (20.0)
Multiracial^a^	1 (10.0)	0
White	7 (70.0)	7 (70.0)
Blood type		
A	3 (30.0)	2 (20.0)
O	7 (70.0)	8 (80.0)
BMI, mean (SD)	28.8 (4.6)	27.6 (7.5)
Diagnosis		
Ischemic cardiomyopathy	1 (10.0)	NA
Idiopathic dilated cardiomyopathy	7 (70.0)	NA
Congenital cardiomyopathy	1 (10.0)	NA
Familial cardiomyopathy	1 (10.0)	NA
Cause of death		
Drug overdose	NA	3 (30.0)
Seizure	NA	1 (10.0)
Asphyxiation	NA	4 (40.0)
Trauma	NA	2 (20.0)
Listing status		
1	2 (20.0)	NA
2	6 (60.0)	NA
4	2 (20.0)	NA
Perfusion times, mean (SD), min		
Cold Ischemia time	40.4 (10.2)	NA
Warm Ischemia time	20.9 (5.2)	NA
Total machine perfusion time	355.0 (91.6)	NA

Abbreviations: BMI, body mass index (calculated as weight in kilograms divided by height in meters squared); NA, not applicable.

^a^ Multiracial refers to a self-reported designation, without further subclassification from the patient.

The mean (SD) warm ischemic time was 20.9 (5.2) minutes, representing the agonal period until ice was placed into the donor chest and cardioplegia delivered to the root during procurement. The mean (SD) cold ischemia time was 40.4 (10.2) minutes, representing the time from ice in the chest of the donor to perfusion on the ex vivo organ perfusion platform. The mean (SD) total machine perfusion time was 355 (91.6) minutes, representing time from initiation of machine perfusion to implantation in the recipient. The mean (SD) recipient cardiopulmonary bypass time was 201.2 (69.1) minutes and mean (SD) cross clamp time was 88.7 (42.4) minutes. All patients left the operating room receiving epinephrine and inhaled nitric oxide, which is standard practice at our institution for heart transplant recipients.

The median (range) follow-up was 97.5 (14.0-293.0) days, with 10 patients (100%) discharged home. Ten of 10 patients (100%) survived, and no patients required postoperative initiation of ECMO. No patients required IABP or RVAD placement postoperatively. All patients have been discharged from the hospital with a median (IQR) length of stay of 29.0 (24.0-35.0) days, and a mean (SD) length of stay of 29.9 (12.8) days. The median (range) intensive care unit (ICU) length of stay was 11.5 (3.0-33.0) days, and the mean (SD) ICU length of stay was 10.6 (4.7) days. There were no episodes of acute rejection necessitating immunosuppression modification posttransplant prior to discharge in any of our recipients.

## Discussion

In this case series, we performed 10 consecutive DCD heart transplants using a beating heart implantation method with 100% survival and no use of postoperative ECMO for graft dysfunction nor any other forms of postoperative mechanical circulatory support. The beating heart method involves cannulation of the donor heart while it is perfused from the perfusion platform and then switching to perfusion from the recipient cardiopulmonary bypass circuit in an uninterrupted fashion. Then, implantation is performed with the allograft beating, removing the second arrest and, with it, the warm and cold ischemic periods typically associated with conventional DCD heart transplantation.

While a noninferiority trial of DCD heart transplantation has shown comparable 6-month survival, 18 of 80 patients (22.5%) who underwent DCD heart transplantation experienced moderate to severe primary graft dysfunction.^10^ In a national, multicenter retrospective study^6^ of outcomes after DCD heart transplantation, the rate of ECMO initiation was 40% postoperatively in the 50 patients who underwent DCD heart transplantation compared with 16% in the 179 patients in the brain death donor cohort. Furthermore, a UK-based 5-year cohort study^11^ of 79 DCD heart transplant recipients showed a 32% incidence of postoperative IABP use. Because no patient in our series required initiation of mechanical circulatory support, including postoperative ECMO and IABP, we hypothesize that the heart was protected from a second bout of ischemia reperfusion injury because of early uninterrupted reperfusion and avoidance of the second cardioplegic arrest. This may contribute to the elevated rates of primary graft dysfunction seen in DCD heart transplantation.^12^

In traditional DCD heart transplantation, a question arises on whether the first of the 2 ischemic periods mitigates the ischemia reperfusion injury of the second via ischemic preconditioning (IPC).^13^ Animal-based investigations of IPC suggest that a short period of ischemia followed by reperfusion preceding a longer period of ischemia reduces the injury pattern of ischemia reperfusion injury.^14^ However, in DCD heart transplantation, the first period of ischemia associated with donor withdrawal, arrest, and cannulation for machine perfusion far exceeds the short periods of ischemia typically described in IPC. In our study, the mean cold ischemic time neared 41 minutes, and the mean warm ischemic time was 21 minutes. Thus, we hypothesize that the benefit of removing an entire period of ischemia from DCD heart transplantation outweighs the potential benefit of ischemic preconditioning associated with 2 periods of ischemia and reperfusion.

We hope these promising early results will inspire further adoption and evaluation of beating heart transplantation. Our method of beating heart implantation can be further extended to heart transplantation from DBD as well in scenarios where ex vivo heart perfusion may be used, such as in extended distance donation.

### Limitations

This study has limitations. This case series is limited by relative homogeneity of its patient population, given all of the recipients were male and most were White individuals.

## Conclusions

This case series found that the beating heart transplantation was successful in 10 of 10 patients, with no initiation of postoperative mechanical circulatory support. Uninterrupted coronary perfusion through implantation may protect the heart from the additional ischemia reperfusion injury typically associated with DCD heart transplantation compared with brain death donation, which may contribute to the rates of postoperative mechanical circulatory support use after DCD heart transplantation.

## Notes

Supplement 1. **eFigure.** Illustrations of Key Steps in Beating Heart Transplantation

Supplement 2.
Data Sharing Statement
