# Errors in Reporting of Ethical Approval and Informed Consent

**DOI:** 10.1001/jamanetworkopen.2026.4862

**Published:** 2026-03-05

In the Original Investigation titled “Beating Heart Transplant Procedures Using Organs From Donors With Circulatory Death,”^1^ published March 11, 2024, the authors had incorrectly reported that the Stanford University institutional review board had approved this study and the participants had provided informed consent. The correct statements are: “This case series was deemed exempt from approval by the Stanford University institutional review board. The exemption for data use for this secondary analysis did not require informed consent.” This article has been corrected.^1^
