# Supplementary material for: Beating Heart Transplant Procedures Using Organs From Donors With Circulatory Death
Source: JAMA Netw Open. 2024 Mar 11;7(3):e241828. doi: 10.1001/jamanetworkopen.2024.1828 (PMC10928498; doi:10.1001/jamanetworkopen.2024.1828)
Supplement: Supplement 1. — eFigure. Illustrations of Key Steps in Beating Heart Transplantation [file jamanetwopen-e241828-s001.pdf]

Supplemental Online Content

Krishnan A, Ruaengsri C, Guenthart BA, et al. Outcomes of beating heart transplant procedures using organs from donors with circulatory death. *JAMA Netw Open*. 2024;7(3):e241828. doi:10.1001/jamanetworkopen.2024.1828

eFigure. Illustrations of Key Steps in Beating Heart Transplant

This supplemental material has been provided by the authors to give readers additional information about their work.

© 2024 et al. *JAMA Network Open.*

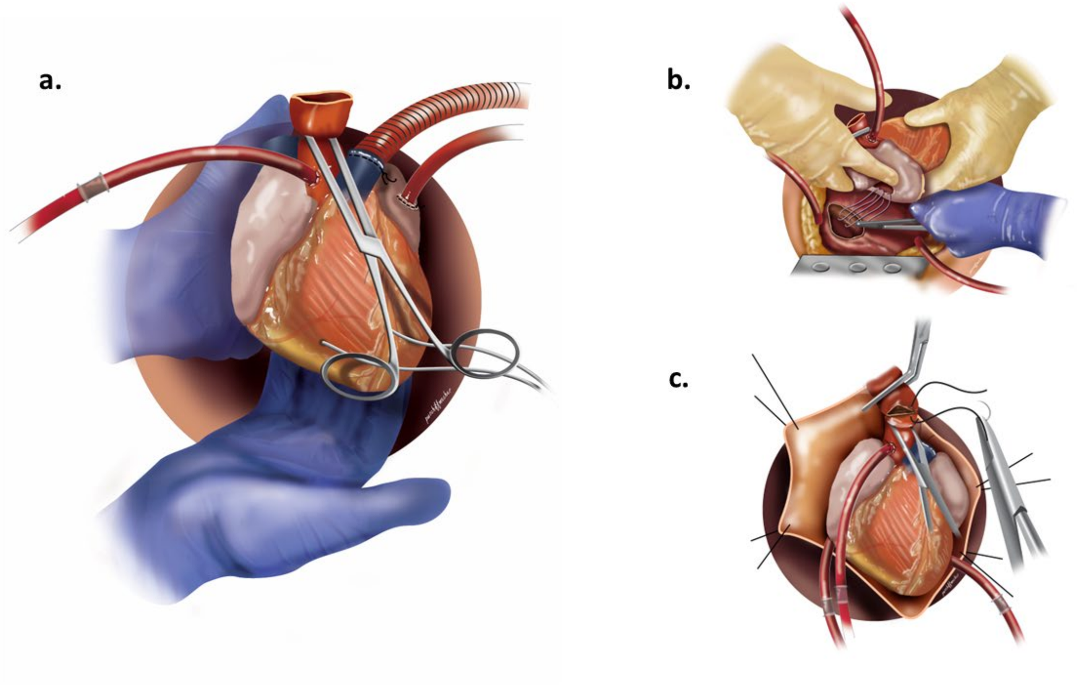

eFigure. Illustrations of Key Steps in Beating Heart Transplantation

Beating heart transplantation is performed by connecting the donor heart to the recipient cardiopulmonary bypass circuit while it is being perfused by the ex vivo organ perfusion platform. Key steps are shown here. In panel A. the preparations for uninterrupted perfusion are shown, including an additional cross clamp, a cannula in the aortic root to deliver warm blood from the cardiopulmonary bypass circuit, a left ventricular vent, and a pulmonary artery vent. Panel B. demonstrates the assistant holding the beating heart while the surgeon sews in the left atrial cuff. Panel C. shows the aortic anastomosis, which is performed between two cross clamps.

© 2024 et al. *JAMA Network Open.*
