# Supplementary material for: Beating Heart Transplant Procedures Using Organs From Donors With Circulatory Death
Source: JAMA Netw Open. 2024 Mar 11;7(3):e241828. doi: 10.1001/jamanetworkopen.2024.1828 (PMC10928498; doi:10.1001/jamanetworkopen.2024.1828)
Supplement: Supplement 2. — Data Sharing Statement [file jamanetwopen-e241828-s002.pdf]

Data Sharing Statement

Krishnan. Outcomes of Beating Heart Transplant Procedures Using Organs From Donors With Circulatory Death. *JAMA Netw Open*. Published March 11, 2024.
doi:10.1001/jamanetworkopen.2024.1828

Data
Data available: No

Additional Information
Explanation for why data not available: Given that this is a 10 patient case series, there is too much identifying information available in the raw data.
